# Outcomes of Warfarin Home INR Monitoring vs Office-Based Monitoring: a Retrospective Claims-Based Analysis

**DOI:** 10.1007/s11606-023-08348-4

**Published:** 2023-12-15

**Authors:** Andrea Van Beek, Mariola Moeyaert, Bishoy Ragheb, Erika Price, Joanna P. MacEwan, Naseeruddin Ahmed, Jack Ansell

**Affiliations:** 1 Visalia Medical Clinic, Visalia, CA USA; 2 University at Albany – State University of New York, Albany, NY USA; 3 VA Eastern Colorado Health Care System, Aurora, CO USA; 4 San Francisco VA Health Care System, University of California San Francisco, San Francisco, CA USA; 5 Genesis Research, Hoboken, NJ USA; 6 Clinical & Medical Affairs Abbott Laboratories, Abbot Park, IL USA; 7 Hofstra Northwell Zucker School of Medicine, Hempstead, NY USA; 8 Long Branch, NJ USA

**Keywords:** warfarin, patient self-testing, INR, prothrombin time, point of care

## Abstract

**Background:**

Home INR testing (patient self-testing) is feasible and effective for warfarin patients but little is known about real-world differences in outcomes for patients using PST versus laboratory-based INR monitoring.

**Objective:**

To compare the safety/efficacy of patient self-testing of real-world warfarin therapy versus office/lab-based monitoring of therapy.

**Design/Setting/Participants/Exposure:**

A retrospective claims-based analysis of warfarin patients enrolled in the MarketScan® Commercial Claims and Encounters and Medicare databases between January 1, 2013, and March 30, 2020. Stratification was based on INR testing method: patient self-testing versus testing at physicians’ offices/local laboratory. The probability of adverse events in each cohort was determined after adjusting for demographic and baseline clinical characteristics using a repeated measures analysis.

**Main Measures:**

Rates of all adverse events: deep venous thrombosis, pulmonary embolism, bleeding, and stroke. A secondary outcome of interest was emergency department visits.

**Key Results:**

A total of 37,837 patients were included in the analysis: 1592 patients in the patient self-testing group and 36,245 in the office-based therapy group. After adjusting for demographic and baseline clinical characteristics, patients in the office-based group had statistically significantly higher rates of all adverse events (incidence rate ratio [IRR]=2.07, 95% CI [1.82, 2.36]), and specific adverse events including thromboembolism (IRR=4.38, 95% CI [3.29, 5.84]), major bleed (IRR=1.45, 95% CI [1.28, 1.64]), and stroke (IRR=1.30, 95% CI [1.05, 1.61]) than patients in the patient self-testing group. Office-based patients also had a statistically significant higher rate of emergency department visits than patient self-testing patients (IRR = 1.65, 95% CI [1.47, 1.84]).

**Conclusions/Relevance:**

This analysis of real-world claims data shows lower rates of stroke, thromboembolism, and major bleeding, as well as fewer emergency department visits, with patient self-testing compared to office-based/lab INR monitoring. Our finding that PST is safe and effective among current users suggests that more patients may benefit from its use.

**Supplementary Information:**

The online version contains supplementary material available at 10.1007/s11606-023-08348-4.

## INTRODUCTION

Warfarin was the mainstay of oral anticoagulation for over 60 years until the introduction of direct oral anticoagulants in 2010. Around 2 million Americans still receive warfarin with many more worldwide,^1^ and warfarin remains the primary anticoagulant of choice for several medical indications. However, warfarin therapy presents many challenges with numerous factors influencing its effectiveness. Frequent INR testing is needed to ensure optimal dosing, and to minimize the risk of bleeding or thromboembolic events.^2^ High-quality warfarin management has been shown to improve the safety and efficacy of the medication, leading to improved patient outcomes.^3^

Warfarin management is complex and labor intensive. Historically, patients had INR testing performed in a physician’s office or laboratory. This required that patients travel to testing sites, which can be challenging for patients who live in rural settings, have mobility or transportation issues, travel often, and/or have extended working hours. Limited access to care during the COVID pandemic added to these challenges.^4^

Point of care coagulometers allow for INR measurement at home. This is done through patient self-testing (PST) programs, which provide education and testing equipment. In PST programs, participants’ INR results are interpreted by clinicians who provide recommendations for dose adjustment via telephone or video visit, without requiring an in-person office encounter.

PST has been shown to improve the quality of care by engaging patients in their healthcare, resulting in an improvement in their quality of life.^5^ Additionally, home INR monitoring allows for more frequent monitoring and has been shown to reduce adverse events (AEs), such as bleeding and clotting.^5–7^

Although PST is recommended by clinical guidelines,^8^ it has faced limited acceptance by physicians and limited reimbursement by third-party payers, especially Medicare.^9^ To further assess the value of home INR monitoring, we evaluated outcomes of warfarin therapy from a large database of third-party/administrative claims (Medicare and commercial insurance) and compared the safety and efficacy of warfarin therapy with INR measurements obtained by PST versus office-based monitoring of therapy.

## METHODS

A retrospective claims-based analysis of patients on warfarin therapy was conducted using MarketScan® Commercial Claims and Encounters (CCAE) and Medicare databases between January 1, 2013, and March 30, 2020. The primary outcomes of interest were the rate of any adverse event (AE) including thromboembolism, stroke, or bleeding, and the rates of each individual category of AE. Patients were stratified based on INR testing method: (1) patients using PST of INR and (2) patients tested at their physician’s office by point of care (POC) testing or at a local laboratory (POC/Lab). Stratification was based on INR testing method using Current Procedural Terminology (CPT) codes for home-based INR testing (categorized as PST), versus CPT codes that did not specify a location and Logical Observation Identifier Names and Codes (LOINC) for INR testing, which were categorized as office-based testing either using point of care devices or laboratory testing).

### Inclusion and Exclusion Criteria

Patients over 18 years of age with (i) a diagnosis (≥ 1 inpatient claim or ≥ 2 outpatient claims) of atrial fibrillation (AF), deep venous thrombosis (DVT), mechanical heart valve (MHV), or pulmonary embolism (PE) between January 1, 2013, and March 31, 2020 (diagnosis date); (ii) at least 1 warfarin claim within the 30-day period including and following the diagnosis date (index date) and no gap in warfarin treatment (days’ supply of medication) of > 30 days; (iii) at least 6 months of continuous insurance plan enrollment prior to the diagnosis date; (iv) continuous insurance plan enrollment between the initial diagnosis date and index date; (v) at least 6 months of continuous enrollment following the index date; and (vi) no claims for a direct acting oral anticoagulant (DOAC) within 6 months of the index date were included in the analysis.

### Variables

The following patient demographic and clinical variables were described: age, sex, geographic region, region location (rural or urban), comorbidities of interest (hypertension, heart failure, valvular disease, diabetes, chronic obstructive pulmonary disease, renal disease, peripheral vascular disease, cancer, obesity, and Charlson Comorbidity Index), health insurance type (Commercial, Medicare), index diagnosis, diagnosis year, and hospital admissions or emergency department (ED) visits prior to index date. The follow-up period continued until the earliest of (i) end of the study period, (ii) loss of insurance enrollment, (iii) treatment discontinuation, or (iv) switch to DOAC.

Clinical outcomes included the following: (i) arterial or venous thromboembolism, (ii) stroke, (iii) major bleeding, (iv) any AE (i.e., thromboembolism, major bleed, or stroke) all identified by ICD 9/10 diagnosis codes (see appendix). A secondary outcome of interest was emergency department (ED) visits. Patients were censored after their first AE in the model of the “any” AE outcome. For the individual AE models, patients were censored after only that specific AE.

### Analysis

Descriptive statistics were used for demographics, baseline clinical characteristics, and resource use of the patient sample stratified by INR testing location (PST vs POC/Lab). Patients with 50% or more of their testing done at POC/Lab were categorized as POC/Lab and those who had more than 50% of testing as PST were categorized as PST. For continuous variables, the Kruskal-Wallis rank-sum tests were used. For categorical variables, chi-squared tests were used.

The probability of experiencing adverse events in each cohort was determined after adjusting for patients’ demographic and baseline clinical characteristics using a repeated measures analysis, which allows for patients to be exposed to different INR testing locations during the follow-up period as well as adjusting for any time-varying covariates and varying follow-up time. A generalized estimating equation (GEE),^10^ with binomial error distribution and logit link function, was used for the repeated measures analysis. Separate GEE models were estimated for the rates of the five outcomes of interest/AEs (i.e., thromboembolism, major bleed, stroke, any of the AEs, and ER visits) as a function of INR testing location and patients’ demographic and baseline clinical characteristics.

Because patients using PST occasionally have POC/Lab-based INRs and such tests may have been obtained immediately before an AE, we wanted to ensure events were attributed to the correct testing mode. Accordingly, in the repeated measures GEE model, patients were considered to have switched from PST INR testing mode to POC/Lab model only if and when they had at least 3 consecutive POC/Lab INR testing claims. Patients were considered to have switched from POC/Lab to PST testing if and when they had at least 1 PST INR claim.

## RESULTS

**Fig. 1:**
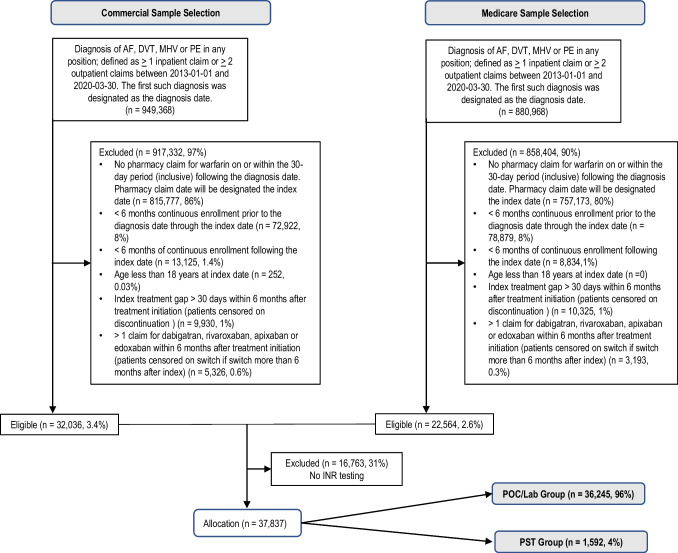
CONSORT flow diagram for same selection and allocation to PST group versus POC/Lab group. Data source: MarketScan Medicare data from 2013-01-01 through 2020-03-30. Abbreviations: AF, atrial fibrillation; DVT, deep vein thrombosis; MHV, mechanical heart failure; PE: pulmonary embolism.^1^: Patients using DOACs were not considered to switch/discontinue if they changed the DOAC medication being used.

A total of 54,600 patient claim records met inclusion criteria (32,036 commercial patients and 22,564 Medicare patients). Of 54,600 patients, 37,837 (69.3%) had INR testing claims and were included in the final analysis. A total of 1592 patients were categorized in the PST group, and 36,245 patients were in the POC/Lab group (Fig. 1, CONSORT flow diagram). For the PST group, the median percentage of total INRs performed using PST was 96.4% (IQR = (77.8%, 100%)) and the mean was 88.1% (SD = 14.8%). For the POC/Lab group, the median percentage was 100% (IQR = (100%, 100%)) and the mean was 99.6% (SD = 3.6%).

### Demographic and Clinical Characteristics of Patient Groups

Table 1 summarizes demographic and clinical characteristics of the two patient groups (PST versus POC/Lab). PST patients were slightly older than POC/Lab patients (median 64 versus 60 years of age at index), and a slightly higher percentage of PST patients vs POC/Lab patients were women (48% versus 44%). There was no difference in proportion of rural vs urban patients between the groups, and similar numbers of patients came from the various regions of the USA.

**Table 1:** Overview of Demographic and Clinical Characteristics by INR Testing Group

	Overall INR testing	PST	POC/Lab	*p*-value	2(df)
	(*N*= 37,837)	(*N*=1592)	(*N*=36,245)		
Demographic characteristics					
Age at diagnosis, years				< .001	92.583 (1)
Mean (SD)	60 (15)	64 (16)	60 (15)		
Median (Q1, Q3)	60 (51, 70)	64 (54, 76)	60 (51, 70)		
Age at treatment index, years				<.001	92.776 (1)
Mean (SD)	60 (15)	64 (16)	60 (15)		
Median (Q1, Q3)	60 (51, 71)	64 (54, 76)	60 (51, 70)		
Sex				<.001	9.0894 (1)
Female	16,874 (45%)	769 (48%)	16,105 (44%)		
Male	20,963 (55%)	823 (52%)	20,140 (56%)		
Region					
Northeast	6786 (18%)	352 (22%)	6434 (18%)	<.001	64.313 (4)
North Central	12,115 (32%)	469 (29%)	11,646 (32%)		
South	12,329 (33%)	588 (37%)	11,741 (32%)		
West	6279 (17%)	170 (11%)	6109 (17%)		
Missing	328 (0.9%)	13 (0.8%)	315 (0.9%)		
Region location				0.200	0.500 (1)
Rural	5904 (16%)	259 (16%)	5645 (16%)		
Urban	31,933 (84%)	1333 (84%)	30,600 (84%)		
Clinical characteristics					
Index diagnosis				<.001	305.620 (4)
Atrial fibrillation	12,160 (32%)	639 (40%)	11,521 (32%)		
Deep vein thrombosis	13,172 (35%)	420 (26%)	12,752 (35%)		
Mechanical heart valve	2657 (7.0%)	255 (16%)	2402 (6.6%)		
Pulmonary embolism	6575 (17%)	181 (11%)	6394 (18%)		
More than 1	3273 (8.7%)	97 (6.1%)	3176 (8.8%)		
Year of diagnosis				<.001	39.586 (6)
2013	7309 (19%)	273 (17%)	7036 (19%)		
2014	11,900 (31%)	451 (28%)	11,449 (32%)		
2015	7678 (20%)	311 (20%)	7367 (20%)		
2016	4894 (13%)	221 (14%)	4673 (13%)		
2017	2968 (7.8%)	163 (10%)	2805 (7.7%)		
2018	1890 (5.0%)	113 (7.1%)	1777 (4.9%)		
2019	1198 (3.2%)	60 (3.8%)	1138 (3.1%)		
Baseline hypertension	21,055 (56%)	966 (61%)	20,089 (55%)	<.001	16.837 (1)
Baseline heart failure	6409 (17%)	385 (24%)	6024 (17%)	<.001	61.466 (1)
Baseline valvular dis	8191 (22%)	632 (40%)	7559 (21%)	<.001	318.130 (1)
Baseline diabetes	9131 (24%)	419 (26%)	8712 (24%)	.110	4.216 (1)
Baseline chronic pulmonary disease	6779 (18%)	326 (20%)	6453 (18%)	<.001	7.232 (1)
Baseline renal disease	3616 (9.6%)	180 (11%)	3436 (9.5%)	<.001	5.677 (1)
Baseline periph vasc dis	4927 (13%)	312 (20%)	4615 (13%)	<.001	62.86 (1)
Baseline cancer	4099 (11%)	138 (8.7%)	3961 (11%)	<.001	7.832 (1)
Baseline obesity	6321 (17%)	214 (13%)	6107 (17%)	<.001	12.478 (1)
Charlson Comorbidity Index				<.001	42.208 (1)
Mean (SD)	1.83 (2.23)	1.98 (2.10)	1.77 (2.22)		
Median (Q1, Q3)	1.00 (0.00, 3.00)	1.00 (0.00, 3.00)	1.00 (0.00, 3.00)		
Payer channel				<.001	213.27 (1)
Commercial	25,267 (67%)	794 (50%)	24,473 (68%)		
Medicare	12,570 (33%)	798 (50%)	11,772 (32%)		

Regarding clinical characteristics, more PST patients had atrial fibrillation and mechanical heart valve as the index diagnosis (40% versus 32%; 16% versus 6.6%), and fewer had deep vein thrombosis or pulmonary embolism (26% versus 35%; 11% versus 18%) (*p*<0.001 for differences across categories). PST patients also had higher rates of baseline comorbidities including hypertension, heart failure, valvular disease, chronic obstructive pulmonary disease, chronic kidney disease, and peripheral vascular disease than patients in the POC/Lab group. POC/Lab patients were more likely to have cancer and obesity. These differences were all small in absolute magnitude, but statistically significant. PST patients had a slightly higher Charlson Comorbidity Index scores than POC/Lab patients (1.98 vs 1.77, *p* < 0.001). The PST patients were evenly divided between commercial insurance and Medicare, whereas 68% of the POC/Lab patients had commercial insurance and 32% had Medicare.

### Adverse Events

#### Descriptive Statistics

Table 2 summarizes the unadjusted estimates of the number of AEs (per 100 patient-years) for all AEs, thromboembolism, major bleed, and stroke. For all AEs, the number of AEs per 100 patient-years for the POC/Lab group is more than 1.7 times higher than that for the PST group. Regardless of gender, age, location, index diagnosis, and payer, the number of AEs for POC/Lab is consistently larger than that for PST (ranging from 1.2 times higher for PE as the index diagnosis to 3.0 times higher for MHV as the index diagnosis).

**Table 2:** Adverse Events per 100 Patient-Years by Testing Location (Unadjusted)

	Any event		Thromboembolism		Major bleed		Stroke	
	PST	POC/Lab	PST	POC/Lab	PST	POC/Lab	PST	POC/Lab
All patients	23.7	40.9	6.85	15.2	15.0	24.7	5.22	6.16
Gender								
Female	28.9	44.4	9.07	17.4	19.0	27.5	5.3	6.12
Male	19.4	38.3	4.97	13.6	11.7	22.6	5.15	6.19
Age								
<65	20.8	40.3	7.22	17.9	11.1	22.3	3.42	4.18
≥65	27.4	42.3	6.35	9.54	20.2	30.2	7.78	10.9
Location								
Urban	22.9	40.9	6.66	15.3	14.6	24.9	5.27	6.15
Rural	27.5	40.6	7.86	14.9	16.8	23.5	4.98	6.21
Index diagnosis								
AF	21.8	34.6	1.32	2.83	16.8	25.7	7.58	9.66
DVT	32.4	48.1	15.30	23	15.6	23.1	5.49	4.76
MHV	11.2	34.1	0.765	1.28	8.7	27.4	2.44	7.02
PE	38.0	43.9	23.8	31.3	14.4	25.3	3.55	3.92
Payer								
Commercial	20.5	40.1	6.83	18.0	11.4	22.2	3.24	4.18
Medicare	27.6	42.3	6.88	9.57	19.6	30.4	7.9	10.9

#### Repeated Measures Analysis using GEE

**Fig. 2:**
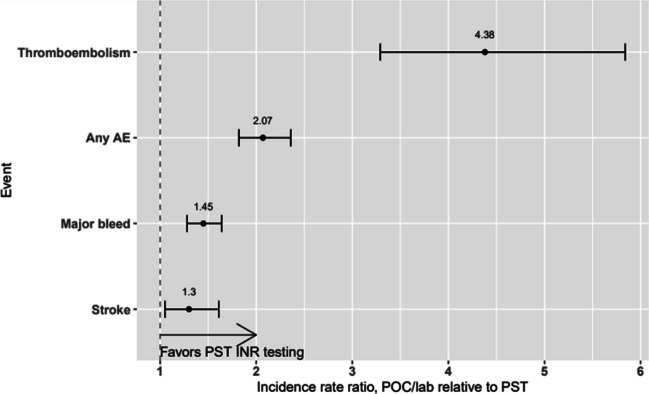
Forest plot of adjusted odds ratio for POC/Lab relative to PST per type of event**.**

The results of the repeated measures regression analysis are presented in Table 3 with a side by side summary of the results for any type of AE, and each of the three types of AEs (i.e., thromboembolism, major bleed, and stroke). Any incidence rate ratio (IRR) larger than 1.0 indicates that the incidence of AEs versus no adverse events is higher in the POC/Lab group relative to the PST reference group, controlling for demographic and clinical baseline variables (the remaining rows in the table). As demonstrated in Table 3, all the IRRs for POC/Lab testing are larger than 1.0 and are statistically significant. Regardless of the type of AE and patient demographic and clinical characteristic, the rate of AEs among patients in the POC/Lab group was always statistically significantly larger than that among patients in the PST reference group. The IRR for POC/Lab testing location was largest for thromboembolism (IRR: 4.38, 95% CI: [3.29, 5.84]) followed by any AE (IRR = 2.07, 95% CI [1.82, 2.36]), major bleed (IRR = 1.45, 95% CI [1.28, 1.64]), and stroke (IRR = 1.30, 95% CI [1.05, 1.61]). Figure 2 shows a forest plot of IRR by event type.

**Table 3:** Repeated Measures Analyses for Any Adverse Event (Thromboembolism, Major Bleed, or Stroke)

Variable	Reference level	IRR [95% CI]			
		Any event	Thromboembolism	Major bleed	Stroke
Testing site POC/Lab	PST	2.07* [1.82, 2.36]	4.38* [3.29, 5.84]	1.45* [2.28, 1.64]	1.30* [1.05, 1.61]
Age at index	NA	1.00* [0.99, 1.00]	0.99* [0.99, 0.99]	0.99* [0.99, 1.00]	1.02* [1.01, 1.02]
Payer channel Medicare	Commercial	1.08* [1.01, 1.15]	0.74* [0.67, 0.81]	1.36* [1.27, 1.45]	1.48* [ 1.30, 2.68]
Male	Female	0.86* [0.83, 0.89]	0.83* [0.79, 0.88]	0.81* [0.78, 0.85]	0.97 [0.90, 1.04]
Hypertension	No Hypertension	1.05* [1.01, 1.09]	0.9* [0.85, 0.96]	1.15* [1.08, 1.21]	1.35* [1.24, 1.47]
Heart failure	No heart failure	1.07* [1.01, 1.12]	0.77* [0.70, 0.84]	1.15* [1.08, 1.21]	1.18* [1.08, 1.30]
Valvular disease	No valvular disease	0.85* [0.81, 0.90]	0.31* [0.28, 0.34]	1.09* [1.04, 1.15]	1.25* [1.15, 1.36]
Diabetes	No diabetes	1.03 [0.99, 1.08]	0.95 [ 0.89, 1.02]	1.06* [1.01, 1.12]	1.27* [1.17, 1.38]
Chronic pulm disease	No chronic pulm disease	1.09* [1.04, 1.14]	1.13* [1.05, 1.22]	1.15* [1.09, 1.21]	1.06 [0.97, 1.16]
Renal disease	No renal disease	1.34* [1.25, 1.42]	1.07 [0.97, 1.19]	1.47* [1.38, 1.57]	1.11 [1.00, 1.24]
Peripheral vascular disease	Peripheral vascular dis	1.21* [1.15, 1.28]	1.24* [1.13, 1.36]	1.19* [1.12, 1.26]	1.52* [1.38, 1.66]
Cancer	No cancer	1.26* [1.19, 1.34]	1.32* [1.21, 1.44]	1.38* [1.30, 1.47]	0.95 [0.85, 1.07]
Obesity	No obesity	0.92* [0.87, 0.97]	0.9* [0.83, 0.97]	1.03 [0.98, 1.09]	0.68* [0.61, 0.76]

*IRR*, incidence rate ratio. Statistically significant effects (i.e.*, p* < .05) are indicated by *. Age at index included as a continuous variable

### Emergency Department Visits of Warfarin Patients Using INR Testing by Testing Location

Controlling for demographic and clinical variables, patients whose testing was primarily POC/lab had a statistically significant higher incidence ratio rate of ED visits than patients with PST (IRR = 1.65, 95% CI [1.47, 1.84]) (Table 4).

**Table 4:** Repeated Measures Analysis for Emergency Department Visits

Variable	Reference level	IRR [95% CI]
Testing site POC/Lab	PST	1.65* [1.47, 1.84]
Age at index	NA	0.99* [0.99, 0.99]
Payer channel Medicare	Commercial	1.39* [1.32, 1.47]
Male	Female	0.81* [0.78, 1.84]
Hypertension	No hypertension	1.11* [1.07, 1.16]
Heart failure	No heart failure	1.14* [1.09, 1.20]
Valvular disease	No valvular disease	0.96* [0.92, 1.00]
Diabetes	No diabetes	1.14* [1.10, 1.19]
Chronic pulmonary disease	No chronic pulmonary disease	1.22* [1.17, 1.28]
Renal disease	No renal disease	1.35* [1.28, 1.43]
Peripheral vascular disease	Peripheral vascular disease	1.11* [1.06, 1.17]
Cancer	No cancer	1.26* [1.19, 1.33]
Obesity	No obesity	1.15* [1.10, 1.20]

*IRR*, incidence rate ratio. Statistically significant effects (i.e., *p* < .05) are indicated by *. Age at index included as a continuous variable

## DISCUSSION

This study of real-world patients further supports the safety and efficacy of PST for warfarin dose management. The cohorts reflected a mostly older population relatively evenly split between male and female patients, and evenly spread across the USA with about 16% from rural locations. Patients in the PST cohort were more likely to have AF or MHV as their index diagnosis, which is not surprising given the older population and long-term requirement for anticoagulation compared to those with VTE. Surprisingly, PST patients had slightly but statistically significantly higher proportions of many comorbidities suggesting that this patient population was a “sicker” cohort as reflected in the slightly higher Charlson Comorbidity Index.

In all categories of the unadjusted analysis except geographic location (demographic, diagnosis, payer), the PST cohort performed better for the outcomes of thromboembolism, major bleed, stroke, and any AE. In the repeated measures analysis controlling for demographic and clinical variables, the PST group consistently showed substantially better outcomes compared with the POC/Lab group. The magnitude of the protective effect of PST was largest for VTE as an adverse event. This may, in part, be due to a higher number of VTE patients in the POC/Lab group. Not surprisingly, patients with greater age at index, Medicare insurance, valvular heart disease, and renal impairment experienced more adverse outcomes regardless of testing site. As a possible reflection of greater utilization of health resources, the office/lab-based cohort had more ED visits compared to the PST cohort. This was also true for patients with Medicare insurance and heart failure.

PST was first approved for Medicare reimbursement in 2001 for patients with MHV^11^ and later (2008) for those with AF and VTE.^12^ Many studies, predominantly retrospective, before and after design, or prospective cohorts have shown PST and patient self-management (PSM where patients also manage their own dose adjustments) of warfarin dosing to be effective in reducing thromboembolism or major bleeding. An individual patient data meta-analysis of eleven studies by Henneghan et al.^6^ in 2012 showed a non-significant reduction in thrombosis (hazard ratio (HR) = 0.74, 95% CI [0.30, 1.82]), major bleeding (HR = 0.84, 95% CI [0.64, 1.12]), or death (HR = 0.94, 95% CI [0.75–1.11]) in patients performing self-testing alone whereas patients who performed self-dose management achieved a significant reduction in thrombosis. Another meta-analysis of 22 trials, Bloomfield et al.^5^ found that patients assigned to either PSM or PST had a significant reduction in risk of death (OR = 0.74, 95% CI [0.63, 0.87]), a significant reduction in risk of major thromboembolism (OR = 0.58, 95% CI [0.45, 0.75]), and no increased risk for major bleeding compared to patients managed by an anticoagulation clinic or usual care. A Cochrane Systematic Review of 28 trials (2016) revealed that PST significantly reduced major thromboembolism (relative risk (RR) = 0.69, 95% CI [0.49, 0.97]) but not major hemorrhage or death.^13^ PSM did produce a significant reduction in death, but not major hemorrhage. Most of the trials in this analysis reported improved patient satisfaction and quality of life with PST or PSM. In one of the few randomized trials, Matchar et al.^14^ randomized patients to PST or testing in an anticoagulation clinic. Both groups had similar risk of thrombosis, major bleeding, and death. The PST group had a small but significant improvement in time in therapeutic range (TTR) and patient satisfaction. This randomized trial supports the notion that high-quality warfarin dose management is essential for good outcomes regardless of where testing is done. Lastly, patient self-testing is also recommended as a clinical practice guideline by expert consensus panels including the American College of Chest Physicians,^15^ the American Society of Hematology^8^, and other expert consensus groups.^16^

The current study has several strengths. It represents a real-world population of patients from both rural and urban communities. Although the cohort of PST patients is much smaller than its comparator at only 4% of the total, it is not much different from estimates of PST utilization based on industry statistics which suggest about 7% of patients taking warfarin use PST.^17^ And though the two cohorts are significantly different in terms of their clinical characteristics, by using a repeated measures analysis, these differences are accounted for in the comparison of AE rates.

The study has limitations as well. Our final sample size for patients on warfarin was much smaller than the initial number of patients with claims for relevant diagnoses. This may in part be due to the large and increasing number of patients receiving direct anticoagulant agents over time. It may also be due to patients obtaining warfarin without insurance claims, as warfarin may be obtained at low cost in the community. In addition, there is a strong possibility of selection bias with physicians referring only the most stable and compliant patients for PST home monitoring. It is also possible that clinics offering PST were better resourced overall, and that confounders such as health literacy and socioeconomic status affected both probability of PST referral and likelihood of the outcomes we examined.

Our study was based on ICD-10 coding, and clinicians may code differently in ways that affected our results. We also note that our study included only insured patients.

Despite these limitations, the PST cohort overall had more comorbidities and a slightly higher Charlson Comorbidity Index than the POC/Lab-based cohort, and differences in patient demographic and clinical characteristics were adjusted for in the repeated measures analysis. Although there is a risk of misclassification with higher-risk PST patients being reassigned to the POC/Lab group if they required medical attention for any reason, we limited this risk by requiring that patients have at least 3 consecutive POC/Lab INRs to be re-classified into the POC/Lab group in the repeated measures analysis. Additionally, POC/Lab INRs performed on the same day as an AE were not included in the count.

What might account for the observed performance of the PST group? The frequency of INR testing has been shown to improve TTR and outcomes; more INRs may have been performed by the PST group since testing is generally recommended once weekly, but we could not directly test this hypothesis (claims are bundled for a number of INR tests but do not include the actual number of tests performed). More frequent testing has been shown to improve TTR and could be a factor leading to improved outcomes.^7,16^ It is also possible that PST engages patients in self-care leading to patient empowerment and improved outcomes as has been demonstrated in other chronic conditions.^18^ PST may be a motivational force in terms of medication adherence and other dietary and behavioral factors affecting the outcomes we examined. As noted above, clinic-level support of PST, as well as individual clinicians’ skill or comfort with warfarin dose management, may also lead to better INR control and lower risk of AEs in PST patients.

## CONCLUSION

This analysis of real-world, claims-based data shows lower rates of stroke, thromboembolism, major bleeding, and any AEs in patients who perform INR monitoring at home compared to INR testing in a physician’s office or laboratory. Such testing has been recommended by expert panels since at least 2008. Despite the advent of DOACs, a substantial number of patients continue to receive warfarin. Our finding that PST is safe and effective among current users suggests that more patients may benefit from its use.

### Supplementary Information

ESM 1 (XLSX 469 kb)

## Data Availability

The datasets generated during and/or analyzed during the current study are licensed by Abbott Diagnostics and will be made available upon request.
